# Unmet needs of 1210 Chinese breast cancer survivors and associated factors: a multicentre cross-sectional study

**DOI:** 10.1186/s12885-022-09224-w

**Published:** 2022-02-03

**Authors:** Xiaofan Bu, Cai Jin, Rongrong Fan, Andy S. K. Cheng, Peter H. F. Ng, Yimin Xia, Xiangyu Liu

**Affiliations:** 1 Nursing Teaching and Research Section, Hunan Cancer Hospital/The Affiliated Cancer Hospital of Xiangya School of Medicine, Central South University, Changsha, China; 2 Xiangya School of Nursing, Central South University, Changsha, China; 3 Department of Nursing, Hunan Xiangya Stomatological Hospital, Central South University, Changsha, China; 4 Department of Rehabilitation Sciences, The Hong Kong Polytechnic University, Hong Kong, China; 5 Department of Computing, The Hong Kong Polytechnic University, Hong Kong, China; 6 Department of Health Service Center, Hunan Cancer Hospital/The Affiliated Cancer Hospital of Xiangya School of Medicine, Central South University, Changsha, China

**Keywords:** Breast cancer survivors, Unmet needs, Quality of life, Cross-sectional survey

## Abstract

**Background:**

Breast cancer survivors (BCSs) often have potential unmet needs. Identification of the specific needs of BCSs is very significant for medical service provision. This study aimed to (1) investigate the unmet needs and quality of life (QoL) of BCSs in China, (2) explore the diverse factors associated with their unmet needs, and (3) assess the association between their unmet needs and QoL.

**Methods:**

A multicentre, cross-sectional survey was administered to 1210 Chinese BCSs. The Cancer Survivor Profile-Breast Cancer and the Functional Assessment of Cancer Therapy-Breast scale were administered to survivors who gave informed consent to participate. Data were analysed using t-test, ANOVA, multiple regression analysis, and Pearson correlations.

**Results:**

The 1192 participants completed questionnaires (response rate 98.51%). Our study reveals that the most prevalent unmet needs were in the ‘symptom burden domain’. The unmet needs of BCSs depend on eleven factors; age, time since diagnosis, education level, occupation, payment, family income status, stage of cancer, treatment, family history of cancer, pain, and physical activities. To ensure the provision of high-quality survivorship care and a high satisfaction level, more attention should be paid to actively identifying and addressing the unmet needs of BCSs. The problem areas identified in the Cancer Survivor Profile for breast cancer were negatively associated with all subscales of QoL except the health behaviour domain, with the correlation coefficient ranging from − 0.815 to − 0.011.

**Conclusion:**

Chinese BCSs exhibit a high demand for unmet needs in this study, and the most prevalent unmet needs were in the ‘symptom burden domain’. There was a significant association between patients’ unmet needs (as defined in the Cancer Survivor Profile for breast cancer) and QoL. Future research should focus on enhancements to survivorship or follow-up care to address unmet needs and further improve QoL.

## Introduction

Female breast cancer is currently the most common type of cancer experienced by women worldwide, with an estimated 2.3 million new cases in 2020 [1]. The five-year relative survival rate for individuals with breast cancer is 82% [2]. Early detection and diagnosis, multimodal therapies, and continuing advances in treatment efficacy have greatly improved survival rates. However, this positive development entails survivors having disease- and treatment-related unmet needs [3]. Therefore, more attention must be paid to the unmet needs of breast cancer patients and to their quality of life (QoL) during and after treatment. The majority of breast cancer survivors (BCSs) reported informational, psychological, physical, and social support needs [4–6]. The prevalence of unmet needs among BCSs varied across studies. Approximately 44% -93% of BCSs reported at least one unmet need [7–13].

Information provision refers to information provided by healthcare providers/nurses in oral, written, or other form [14]. A systematic review indicated that patients with fulfilled information needs and patients who experience fewer information barriers have better QoL and lower levels of depression and anxiety [14]. Informational needs were positively associated with future levels of anxiety as well [15]. Healthcare providers should pay more attention to patient-centred information provision. The diagnosis and treatment of breast cancer results in considerable psychological consequences, and breast cancer may remain unrecognised and untreated. Schmid-Büchi et al. reported that what patients’ most needed help with were psychological issues [16]. This generally stems from the inability of patients to efficiently cope with the psychological burden generated by a cancer diagnosis and treatment. Martínez Arroyo et al. [17] and Skandarajah et al. [18] reported that needs focusing on the possibility of recurrence were the most frequent among BCSs. Fear of cancer recurrence is a near-universal worry for cancer survivors [19]. Persistent high levels of preoccupation or worry and hypervigilance to bodily symptoms presenting for at least 3 months are key characteristics of clinical fear of cancer recurrence [20]. Patients fear or worry that the cancer will return or progress. When fear of cancer recurrence is left unaddressed, it tends to remain stable across disease trajectories over time [19]. Physical unmet needs include primarily sleep difficulty, pain, cognitive impairment, swelling, numbness and paraesthesia of the affected arm or around the affected breast, physical limitations on the affected arm, heart disease, and sexual difficulties. Lack of social support and higher unmet needs are associated with poorer QoL [21].

According to a systematic review conducted by Ho et al., the health-related QoL of breast cancer patients is poorer than the general population in Asia [21]. Additionally, breast cancer patients with comorbidities and who are undergoing chemotherapy, have lower social support, and more unmet needs are associated with poorer QoL [21, 22]. Unmet needs are strong predictors of the QoL of recurrent breast cancer patients. This suggests that the QoL of women with recurrent breast cancer is possibly affected more by unmet needs than their socio-demographic or clinical characteristics [23].

Factors potentially associated with the unmet needs of BCSs have been reported in previous studies, including such sociodemographic characteristics as age [3, 4, 7, 12, 13, 24–26], employment status [4, 26, 27], marital status [12, 13], education [12, 13], and lower income [25]; such psychological problems/symptoms as stress [4], distress [3, 28], fear of recurrence [7, 17] depressive symptoms [13, 28], anxiety [12, 13], cognitive-emotional impacts [17], and suicidal ideation [4]; such clinical characteristics as chemotherapy [3, 29], cancer stage [24], treatment phase [24], length of time since primary surgery [7], receiving endocrine treatment alone, duration since diagnosis [26], being in an advanced stage [26], negative hormone receptor status [28], hormone treatment [27], receiving endocrine treatment alone [29], negative hormone receptor status [28], and comorbidities [13]; and other related factors such as QoL [30, 31], multiplicity [4], life events [13], conflict in interpersonal relations [16], and a lower performance status [26].

In view of the apparent disparities in socio-demographic factors, cultural aspects, racial aspects, healthcare systems, and service provision between countries, unmet needs and the details of how those needs are experienced and communicated are likely to vary. Approximately 11% of worldwide cases of breast cancer occur in China and incidents have increased rapidly in recent decades [32]. However, few studies have ever been conducted to investigate the prevalence of and factors related to the unmet needs of BCSs in Mainland China. To date, the survey tools of most studies have been universal and do not capture the full extent and diverse aspects of breast cancer patients’ unmet needs. To provide appropriate medical services for BCSs, efforts should be made to identify factors associated with unmet needs and the specific characteristics of those unmet needs. To address these gaps, the Hunan Cancer Hospital, in collaboration with nine provincial cancer hospitals, designed and implemented this study. In the present study, we analyse the experiences of BCSs from a comprehensive perspective to identify problems with physical and emotional symptoms, health behaviour, financial strain, and healthcare-seeking skills during and after treatment; explore diverse factors associated with unmet needs; and assess the association between the unmet needs and QoL of BCSs in China. Knowledge of the gaps in care can guide the development and implementation of appropriate medical programs and services to address the broad scope of needs of BCSs.

## Methods

### Procedure and participants

Cluster random sampling was used in this study. First, all cancer hospitals in China are categorised geographically as eastern, western, southern, northern, or central. Two hospitals were randomly selected from each region, resulting in cancer hospitals from 10 provinces (Hunan, Guangxi, Beijing, Jiangxi, Henan, Guizhou, Guangdong, Hebei, Xinjiang, and Zhejiang) being included in the study. The sample size was calculated using G-Power software, version 3.1.9.2 based on a linear multiple regression test with an alpha error of 5%, a power of 95%, and 73 predictors in the model. The required sample size was 384. Accounting for invalid questionnaires, 20% was added to the calculated sample size. The final sample size needed to be larger than 461. All questionnaires were completed using online survey methods and using Questionnaire Star (www.wjx.cn), an electronic data collection tool. A QR code was generated giving participants access to the online questionnaire.

From May 2020 to November 2020, we conducted a cross-sectional survey of 1210 participants who voluntarily agreed to participate in the study at ten cancer hospitals. Patients were eligible if they (a) were female, (b) had been diagnosed with breast cancer, (c) had completed primary therapy (surgery, chemotherapy, and/or radiation), (d) were aged 18 or over, (e) were able to understand all questions, (f) provided informed consent. Patients with mental disorders, hearing disorders, or diseases affecting questionnaire completion were excluded from the study. This study was approved by the Hunan Cancer Hospital Research Ethics Committee (Quick review No. 02 in 2020).

### Measures

A self-developed information sheet was designed to elicit information from participants on demographic and disease-related variables such as age, gender, ethnicity, religion, place of residence, marital status, occupation, employment status, education level, family income status, whether patients are receiving Medicare, stage of cancer, and surgery type. Additionally, the questionnaire battery was comprised of the following two measures.

#### Cancer survivor profile-breast Cancer

To measure the unmet needs experienced by breast cancer patients following treatment, the Cancer Survivor Profile-Breast Cancer (CSPro-BC) was used. The original version of the CSPro-BC comprises 5 domains, 18 subscales, and 73 items [33]. Each item was scored using a five-point Likert-type scale (1 = Strongly disagree, 2 = Disagree, 3 = Neutral, 4 = Totally disagree, 5 = Strongly agree). The 5 domains were symptom burden (6 subscales, 14 items), function (5 subscales, 6 items), health behaviour (3 subscales, 3 items), financial strain (1 subscale, 2 items), and healthcare-seeking (3 subscales, 3 items). The scale has good acceptability, internal consistency, and validity. Cronbach’s alpha coefficient ranged from 0.55 to 0.94 for the 5 subscales.

The CSPro-BC has been translated into Chinese and this version has also been rigorously validated [34]. Forward and backward translation, content confirmation, exploratory and confirmatory factor analysis, test-retest reliability, and internal consistency checking by providers and BCSs led to the final version of the instrument employed in the survey, with 5 domains, 18 subscales, and 71 items. The good internal reliability of the Chinese version of the CSPro-BC was established by Cronbach’s alpha coefficient of 0.65–0.92 for the 5 subscales.

#### Functional assessment of Cancer therapy-breast

To assess the multidimensional QoL in patients with breast cancer, the Functional Assessment of Cancer Therapy-Breast (FACT-B) scale was used. The scale consists of 5 subscales and 36 items assessing patients’ physical well-being, functional well-being, emotional well-being, social/family well-being, and breast cancer-specific concerns. Each item was scored using a five-point Likert-type scale (0 = Not at all, 1 = A little bit, 2 = Somewhat, 3 = Quite a bit, 4 = Very much). Cronbach’s alpha coefficient for the FACT-B total score was 0.90, with five subscales ranging from 0.63 to 0.86 [35]. The FACT-B has been translated into Chinese. The internal consistency of the Cronbach’s alpha coefficient of the Chinese version for the five domains mentioned above ranged from 0.59 to 0.85 [36].

### Data analysis

SPSS version 22.0 was employed to conduct all data analysis. The significance level was set at *P* < 0.05. Descriptive statistics were calculated to describe participant characteristics and to summarise the data.

To determine the factors affecting the CSPro-BC level, univariate analyses (independent sample t-tests and one-way ANOVA) were first conducted to explore differences between the variables in terms of CSPro-BC level. The factors used in these analyses were chosen based on the literature review [3, 4]. Factors shown to be significant were then subjected to multiple regression analysis. T-test and ANOVA were used to compare the scores for each factor according to the demographic and other characteristics of BCSs. Multiple regression analysis was applied to assess the influences of major variables on the level of CSPro-BC. In multiple regression analysis, the need scores for each factor were used as dependent variables, and variables that were found to be statistically significant by univariate analysis were used as independent variables. Pearson correlations were conducted to reveal the correlations between the CSPro-BC and FACT-B.

## Results

### Characteristics of the study population

Table 1 shows the characteristics of our study participants. All but 18 of the 1210 patients invited to participate completed the questionnaires; with 10 took less than 5 minutes to fill in the questionnaire, and 8 provided rather regular answers. The mean patient age was 47.51 years (SD 9.09) and the majority were married (93.3%).

**Table 1:** Characteristics of participants (*N* = 1192)

Variable	Group	N	%
Age	< 40	235	19.71%
	40–49	454	38.09%
	50–59	401	33.64%
	≥ 60	102	8.56%
Time since diagnosis (months)	≤ 12	273	22.9%
	13–24	583	48.9%
	25–60	277	23.2%
	≧ 61	59	5.0%
Marital status	Single	18	1.5%
	Married	1112	93.3%
	Divorced	38	3.2%
	Widowed	21	1.7%
	Cohabitation	3	0.3%
Education level	Primary school	229	19.2%
	Secondary school	516	43.3%
	High school or technical secondary school	202	16.9%
	University	236	19.8%
	Master’s degree or above	9	0.8%
Employment	Employed	297	24.9%
	Unemployed	895	75.1%
Occupation	Unemployed	895	75.1%
	Public institution	163	13.7%
	Privately or individually owned business	34	2.9%
	Worker	23	1.9%
	Farmer	5	0.4%
	Private enterprise	72	6.0%
Place of residence	City	441	37.0%
	Township	329	27.6%
	Village	422	35.4%
Family income status (RMB/month/per person)	<2000	201	16.9%
	2000–4999	359	30.1%
	5000–9999	432	36.2%
	10,000–19,999	183	15.4%
	≥ 20,000	17	1.4%
Payment	Free medical treatment	29	2.4%
	Medical insurance	482	40.4%
	New rural cooperative medical insurance	510	42.8%
	Self-paying	83	7.0%
	Serious disease insurance or commercial insurance	88	7.4%
Stage of breast cancer	I	254	21.3%
	II	567	47.6%
	III	279	23.4%
	IV	92	7.7%
Treatment	Surgery therapy	109	9.2%
	Surgery + chemotherapy	385	32.3%
	Surgery + radiation therapy	14	1.2%
	Surgery + chemotherapy + radiation therapy	211	17.7%
	Surgery + chemotherapy + radiation therapy + hormone therapy	179	15.0%
	Surgery + targeted therapy	41	3.4%
	Others^a^	253	21.2%
Family history of cancer	Yes	63	5.3%
	No	1129	94.7%

Others^a^: any other treatment method or combination of surgery, chemotherapy, radiotherapy, targeted therapy, endocrine therapy, or other treatment methods

### Unmet needs of BCSs

The domains of the top three of unmet needs were symptom burden and healthcare-seeking skills (Table 2). The need that was most unmet was fear of recurrence.

**Table 2:** Unmet needs of participants

Rank	Sub-scales	Score range	Score (Mean ± SD)	Scoring rate	Domains
1	Fear of recurrence	6 ~ 30	20.70 ± 6.37	69.0%	Symptom burden domain
2	Patient-provider communication	6 ~ 30	18.97 ± 5.48	63.2%	Health care–seeking skills domain
3	Body image	3 ~ 15	8.92 ± 3.30	59.5%	Symptom burden domain
4	Health information	4 ~ 20	11.30 ± 5.07	56.5%	Health care–seeking skills domain
5	Social	4 ~ 20	10.61 ± 3.44	53.1%	Function domain
6	Physical activity	2 ~ 8	4.25 ± 1.02	53.1%	Health behavior domain
7	Information acquisition	2 ~ 10	5.21 ± 2.74	52.1%	Health care–seeking skills domain
8	Sleep	4 ~ 20	10.35 ± 3.91	51.8%	Function domain
9	Work	1 ~ 10	5.04 ± 0.88	50.4%	Function domain
10	Healthcare competence	6 ~ 30	14.90 ± 4.43	49.7%	Health care-seeking skills domain
11	Financial strain	4 ~ 20	9.69 ± 4.47	48.5%	Financial strain domain
12	Fatigue	5 ~ 25	10.44 ± 4.78	41.8%	Symptom burden domain
13	Anxiety	4 ~ 20	7.97 ± 3.80	39.9%	Symptom burden domain
14	Cognitive	6 ~ 30	11.84 ± 5.19	39.5%	Function domain
15	Depressive symptoms	4 ~ 20	7.74 ± 3.73	38.7%	Symptom burden domain
16	Sexual	2 ~ 10	3.77 ± 1.56	37.7%	Function domain
17	Pain	5 ~ 25	9.31 ± 4.73	37.2%	Symptom burden domain
18	Diet	2 ~ 10	3.24 ± 1.63	32.4%	Health behavior domain

### Needs by clinicopathological characteristics

The < 40 age group showed higher levels of unmet needs in all domains except healthcare-seeking skills. The ≤12-months-since-diagnosis group showed higher levels of unmet needs in the domains of symptom burden, function, and health behaviour, whereas > 61-months-since-diagnosis group showed higher levels of unmet needs in the domains of financial strain and healthcare-seeking skills. The Stage IV group and the group with a family history of cancer showed higher levels of unmet needs in all domains except health behaviour. The group that had received a combination of surgery, chemotherapy, radiation therapy, and hormone therapy showed higher levels of unmet needs in all domains except health behaviour and healthcare-seeking skills. The group with severe pain and the group with heavy physical activities, showed higher levels of unmet needs in all domains (Table 3).

**Table 3:** Needs by clinicopathological characteristics of participants

Variable	symptom burden	function	health behavior	financial strain	health care-seeking skills	Total
Age						
< 40	70.00 ± 19.96	43.18 ± 8.38	7.83 ± 2.05	10.26 ± 4.53	51.49 ± 12.37	179.99 ± 35.68
40–49	66.63 ± 20.03	42.31 ± 9.05	7.56 ± 2.07	10.17 ± 4.43	50.57 ± 11.98	174.36 ± 38.33
50–59	61.55 ± 22.03	40.24 ± 9.05	7.33 ± 2.24	9.01 ± 4.31	49.58 ± 11.63	163.59 ± 40.38
≥ 60	60.80 ± 22.58	40.31 ± 7.93	7.06 ± 2.18	8.91 ± 4.72	50.16 ± 11.31	162.36 ± 38.23
*p*-value	< 0.001	< 0.001	0.005	< 0.001	0.257	< 0.001
Time since diagnosis(months)						
≤ 12	78.27 ± 18.47	44.03 ± 8.02	8.38 ± 2.73	11.30 ± 4.35	54.14 ± 10.83	192.82 ± 33.23
13–24	59.69 ± 19.39	40.57 ± 8.80	7.22 ± 1.88	9.00 ± 4.33	49.07 ± 11.89	162.00 ± 36.75
25–60	62.21 ± 19.38	40.96 ± 9.36	7.12 ± 1.70	9.01 ± 4.23	48.64 ± 11.49	164.69 ± 38.71
> 61	70.92 ± 23.73	43.78 ± 9.35	7.83 ± 2.21	12.24 ± 4.72	54.25 ± 13.94	184.93 ± 44.95
*p*-value	< 0.001	< 0.001	< 0.001	< 0.001	< 0.001	< 0.001
Stage of breast cancer						
I	59.35 ± 19.69	40.06 ± 8.39	7.61 ± 2.64	8.20 ± 4.29	47.91 ± 11.84	160.02 ± 37.26
II	65.10 ± 20.39	41.97 ± 8.80	7.48 ± 1.96	9.74 ± 4.27	50.28 ± 12.10	171.24 ± 38.20
III	66.88 ± 21.22	41.42 ± 9.17	7.34 ± 2.03	10.36 ± 4.55	51.58 ± 10.83	173.58 ± 39.63
IV	75.37 ± 20.70	44.27 ± 9.56	7.70 ± 2.00	11.96 ± 4.44	54.28 ± 12.43	189.66 ± 39.04
*p*-value	< 0.001	0.001	0.380	< 0.001	< 0.001	< 0.001
Treatment						
Surgery therapy	53.18 ± 17.86	39.38 ± 8.57	7.44 ± 2.39	7.00 ± 3.85	45.81 ± 12.01	150.52 ± 35.13
Surgery + chemotherapy	59.57 ± 20.86	40.38 ± 8.64	7.05 ± 2.09	8.74 ± 4.28	48.69 ± 11.49	160.60 ± 38.12
Surgery + radiation therapy	61.86 ± 22.45	40.64 ± 8.97	8.50 ± 2.14	8.71 ± 4.53	52.36 ± 11.14	169.36 ± 39.36
Surgery + chemotherapy + radiation therapy	69.01 ± 19.83	42.65 ± 8.93	7.65 ± 1.78	10.68 ± 4.31	50.48 ± 12.02	176.82 ± 37.27
Surgery + chemotherapy + radiation therapy + hormone therapy	79.66 ± 16.87	44.71 ± 9.03	8.29 ± 1.80	12.15 ± 4 .03	55.52 ± 11.27	197.15 ± 32.05
Surgery + targeted therapy	77.61 ± 14.28	43.85 ± 9.44	8.54 ± 2.83	11.71 ± 4.09	56.51 ± 11.80	193.80 ± 31.32
Others^a^	63.17 ± 19.02	41.09 ± 8.59	7.27 ± 2.26	9.47 ± 4.37	50.12 ± 11.33	167.84 ± 37.33
*p*-value	< 0.001	< 0.001	< 0.001	< 0.001	< 0.001	< 0.001
Family history of cancer						
Yes	77.49 ± 19.08	44.60 ± 9.92	7.68 ± 2.71	10.92 ± 4.14	55.46 ± 11.67	193.06 ± 37.53
No	64.40 ± 20.71	41.45 ± 8.82	7.48 ± 2.11	9.62 ± 4.48	50.10 ± 11.85	169.58 ± 38.79
*p*-value	< 0.001	0.006	0.469	0.025	< 0.001	< 0.001
Pain						
Mild	65.45 ± 16.75	39.58 ± 8.29	7.08 ± 1.77	8.49 ± 4.10	47.69 ± 11.22	155.96 ± 32.90
Moderate	80.64 ± 13.88	45.51 ± 8.61	8.33 ± 2.23	12.00 ± 4.16	55.57 ± 11.13	198.39 ± 29.62
Severe	95.45 ± 16.86	48.01 ± 8.19	8.69 ± 3.41	13.49 ± 3.64	58.80 ± 10.87	220.51 ± 27.07
*p*-value	< 0.001	< 0.001	< 0.001	< 0.001	< 0.001	< 0.001
Physical activity						
Light	62.95 ± 20.74	41.24 ± 8.85	6.89 ± 1.65	9.37 ± 4.46	49.59 ± 11.69	166.47 ± 38.44
Medium	71.20 ± 19.40	42.55 ± 8.85	9.28 ± 2.23	10.62 ± 44.33	52.53 ± 12.14	183.04 ± 37.19
Heavy	91.50 ± 21.23	51.38 ± 10.06	12.25 ± 5.60	13.38 ± 5.24	65.00 ± 6.50	232.50 ± 33.24
*p*-value	< 0.001	0.001	< 0.001	< 0.001	< 0.001	< 0.001

Others^a^: any other treatment method or combination of surgery, chemotherapy, radiotherapy, targeted therapy, endocrine therapy, or other treatment methods

### Needs by sociodemographic characteristic

The employed group and free medical treatment group were found to have higher levels of unmet needs in the domains of symptom burden, function, health behaviour, and healthcare-seeking skills, but the unemployed group and the new rural cooperative medical insurance group showed higher levels of unmet needs in financial strain. The group with a family income status of < 2000 RMB/month showed higher levels of unmet needs in all domains and the worker and farmer groups showed higher levels of unmet needs in sysptom burden domain and health behavior domain. Regarding the level of needs according to the place of residence, the city group showed higher levels of unmet needs in the domains of symptom burden, health behaviour, and healthcare-seeking skills. Regarding the level of needs according to education level, the high school or technical-secondary school group was found to have higher levels of unmet needs in the domains of function, health behaviour, and financial strain, whereas the master’s degree group was found to have unmet needs in the domains of symptom burden and healthcare-seeking skills. In addition, there was no significant difference in the level of unmet needs according to marital status (Table 4).

**Table 4:** Needs by sociodemographic characteristics of participants

Variable	symptom burden domain	function domain	health behavior domain	financial strain domain	health care-seeking skills domain	Total
Marital status						
Unmarried	65.94 ± 18.29	40.39 ± 7.29	8.00 ± 1.64	10.11 ± 4.66	50.83 ± 15.83	174.72 ± 40.71
Married	64.76 ± 20.80	41.58 ± 8.91	7.46 ± 2.10	9.67 ± 4.48	50.32 ± 11.80	170.29 ± 38.95
Divorced	67.00 ± 18.37	42.03 ± 8.69	7.53 ± 1.80	9.66 ± 3.91	51.11 ± 12.05	174.74 ± 34.31
Widowhood	77.81 ± 25.72	42.10 ± 10.29	8.48 ± 4.37	10.19 ± 4.74	52.24 ± 13.78	186.48 ± 49.81
Cohabitation	67.67 ± 20.60	53.00 ± 5.20	8.33 ± 1.53	11.00 ± 6.25	48.67 ± 10.26	183.67 ± 42.10
*p*-value	0.074	0.248	0.189	0.952	0.943	0.347
Education level						
Primary school	63.67 ± 22.97	41.63 ± 9.33	7.16 ± 2.00	10.28 ± 4.79	50.48 ± 11.64	168.71 ± 41.96
Secondary school	60.22 ± 19.54	40.87 ± 9.05	7.12 ± 2.03	9.07 ± 4.30	48.07 ± 11.11	161.32 ± 37.07
High school or technical secondary school	77.53 ± 18.00	43.76 ± 8.38	8.73 ± 2.46	11.78 ± 3.95	55.60 ± 11.06	194.03 ± 32.26
University	65.90 ± 18.99	41.39 ± 8.46	7.56 ± 1.81	8.70 ± 4.36	50.61 ± 12.81	172.78 ± 37.08
Master degree or above	79.78 ± 21.57	41.44 ± 5.36	7.78 ± 2.22	9.56 ± 2.92	57.56 ± 16.01	196.67 ± 38.83
*p*-value	< 0.001	0.004	< 0.001	< 0.001	< 0.001	< 0.001
Employment status						
Employed	68.52 ± 19.40	42.25 ± 8.79	8.00 ± 2.38	9.10 ± 4.15	51.11 ± 12.73	180.06 ± 36.90
Unemployed	63.95 ± 21.16	41.40 ± 8.94	7.32 ± 2.03	9.89 ± 4.56	50.14 ± 11.60	167.75 ± 39.29
*p*-value	0.001	0.158	< 0.001	0.009	0.223	< 0.001
Occupation						
Unemployed	63.95 ± 21.16	41.40 ± 8.94	7.32 ± 2.03	9.89 ± 4.56	50.14 ± 11.60	167.75 ± 39.29
Public institution	68.72 ± 19.13	42.67 ± 8.90	7.85 ± 2.34	8.83 ± 4.27	50.83 ± 12.72	180.01 ± 36.99
Privately or individually owned business	58.41 ± 20.74	39.59 ± 9.47	7.76 ± 1.46	8.35 ± 4.30	46.03 ± 13.85	161.38 ± 41.24
Worker	77.91 ± 15.71	43.74 ± 8.42	9.48 ± 3.65	10.22 ± 3.12	55.74 ± 12.35	197.13 ± 28.00
Farmer	78.60 ± 28.64	39.20 ± 12.21	9.20 ± 1.92	11.20 ± 5.54	52.40 ± 9.56	191.60 ± 51.21
Private enterprise	69.15 ± 18.09	42.26 ± 8.01	7.90 ± 2.21	9.57 ± 3.94	52.58 ± 11.97	182.72 ± 33.11
*p*-value	< 0.001	0.250	< 0.001	0.038	0.029	< 0.001
Place of residence						
City	68.07 ± 19.63	41.82 ± 8.54	7.88 ± 2.39	9.45 ± 4.16	51.73 ± 12.10	176.44 ± 37.18
Township	60.00 ± 19.66	41.11 ± 9.33	6.97 ± 1.96	9.01 ± 4.32	48.51 ± 11.54	162.05 ± 37.73
Village	65.94 ± 22.20	41.79 ± 8.95	7.49 ± 1.90	10.48 ± 4.78	50.44 ± 11.78	171.78 ± 40.85
*p*-value	< 0.001	0.486	< 0.001	< 0.001	0.001	< 0.001
Family income status(RMB/month/per person)						
<2000	83.70 ± 16.34	46.79 ± 8.06	8.24 ± 2.11	14.05 ± 3.75	56.68 ± 10.50	204.84 ± 28.95
2000–4999	70.07 ± 19.77	42.92 ± 9.03	8.15 ± 2.52	11.82 ± 3.52	53.53 ± 11.48	182.86 ± 36.00
5000–59,999	57.58 ± 17.93	39.45 ± 8.57	6.87 ± 1.76	7.57 ± 3.27	47.55 ± 11.13	155.76 ± 34.45
10,000–19,999	52.48 ± 15.93	38.74 ± 7.61	6.79 ± 1.47	5.78 ± 2.80	43.70 ± 10.64	145.18 ± 29.06
≥ 20,000	66.41 ± 20.15	38.76 ± 4.98	8.18 ± 1.63	9.24 ± 4.52	53.59 ± 10.76	172.94 ± 33.06
*p*-value	< 0.001	< 0.001	< 0.001	< 0.001	< 0.001	< 0.001
Payment						
Free medical treatment	74.62 ± 19.25	42.79 ± 8.56	8.97 ± 3.16	9.59 ± 2.92	53.72 ± 9.04	188.76 ± 30.22
Medical insurance	65.17 ± 20.29	41.41 ± 8.92	7.53 ± 2.29	9.10 ± 4.23	50.57 ± 12.30	171.09 ± 38.81
New rural cooperative medical insurance	68.04 ± 21.49	42.37 ± 9.02	7.65 ± 1.98	10.80 ± 4.62	51.45 ± 11.59	176.02 ± 39.49
Self-paying	58.64 ± 18.22	41.24 ± 8.75	7.12 ± 1.85	9.31 ± 4.26	49.37 ± 11.94	161.94 ± 35.36
Serious disease insurance or commercial insurance	50.48 ± 13.75	38.27 ± 7.63	6.25 ± 1.39	6.88 ± 3.53	43.02 ± 9.25	141.67 ± 27.62
*p*-value	< 0.001	0.002	< 0.001	< 0.001	< 0.001	< 0.001

### Multiple regression analysis by needs

Multiple regression analysis was conducted to determine the influence of each independent variable on the total score for each unmet need. The results are shown in Table 5. The results revealed that age, time since diagnosis, education level, occupation, payment, family income status, stage of cancer, treatment, family history of cancer, pain, and physical activities were associated with unmet needs and overall unmet needs. However, there were no statistically significant differences in the level of unmet needs according to place of residence and employment status. The ≥40 age groups showed a lower level of recognition of needs in the symptom burden, health behavior and financial strain domains. The high school or technical secondary school group showed a higher level of recognition in symptom burdern and health behavior domain. In addition, the payment of serious disease insurance or commercial insurance method had a significant effect on unmet needs in the symptom burdern, health behaviour, and healthcare seeking skills domain. Patients who had family history of cancer were more likely to have unmet symptom burdens. Treatment type was most commonly associated with all domains except health behavior domain. The levels of unmet needs were higher in all domains in the group with serve pain while the unmet needs in symptom burden, function and health behavior were higher in patients with medium and heavy physical activity.

**Table 5:** The result of multiple regression analysis by needs

Variable	symptom burden		function		health behavior		financial strain		healthcare-seeking skills		Total needs	
	β	T	β	T	β	T	β	T	β	T	β	T
Age group (reference: < 40), y												
40–49	−0.088	−3.32^*^	0.035	0.941	−0.094	−2.911^*^	−0.017	−0.593	−0.035	−0.979	−0.082	−2.981^*^
50–59	−0.123	−4.499^*^	0.039	1.019	−0.068	−2.055^*^	−0.08	−2.658^*^	−0.017	−0.459	−0.118	−4.133^*^
≥ 60	−0.097	−4.133^*^	−0.045	−1.353	−0.062	−2.141^*^	−0.046	−1.752*	0.01	0.323	−0.081	−3.286^*^
Time since diagnosis (reference: < 12), months												
13–24	−0.117	−4.347^*^	0.048	1.269	−0.121	−3.671^*^	− 0.029	− 0.964	− 0.015	− 0.409	−0.075	− 2.681^*^
25–60	− 0.068	− 2.578^*^	0.112	3.05^*^	− 0.121	− 3.788^*^	− 0.027	− 0.932	− 0.031	−0.867	− 0.048	−1.739
> 61	−0.024	−1.151	0.05	1.697^*^	−0.011	−0.444	0.042	1.785	0.018	0.639	0.005	0.227
Education level (reference: Primary school)												
Secondary school	−0.015	−0.552	− 0.043	−1.147	0.021	0.633	− 0.047	−1.597	− 0.055	− 1.507	− 0.032	−1.13
High school or technical secondary school	0.077	2.786^*^	−0.027	− 0.689	0.079	2.339^*^	0.019	0.621	0.055	1.463	0.058	2.003^*^
University	0.035	1.14	0.015	0.34	−0.013	− 0.333	− 0.004	− 0.125	0.042	0.997	0.021	0.652
Master degree or above	0.033	1.622	−0.058	−2.014^*^	− 0.003	− 0.107	0.029	1.298	0.068	2.44^*^	0.035	1.658
Employment status (reference: unemployed)												
Employed	–	–	–	–	–	–	–	–	–	–	–	–
Occupation (reference: unemployed)												
Public Institution	0.054	2.388^*^	0	−0.015	0.057	2.05^*^	−0.013	− 0.534	0.018	0.594	0.104	4.389^*^
privately or individually-owned business	−0.014	− 0.721	0.02	0.745	0.037	1.561	−0.013	− 0.593	− 0.026	−0.999	0.004	0.218
Worker	0.036	1.797	−0.009	−0.33	0.068	2.833^*^	−0.036	−1.659	0.019	0.712	0.044	2.112^*^
Farmer	0.013	0.668	0.034	1.246	0.043	1.817	−0.023	−1.079	−0.01	− 0.388	0.002	0.116
Private enterprise	0.043	2.127^*^	−0.011	− 0.38	0.038	1.528	0.019	0.874	0.049	1.782	0.084	3.998^*^
Place of residence (reference: village)												
City	0.033	1.211	0.038	0.987	0.031	0.927	0.001	0.026	0.053	1.404	0.046	1.609
Township	0.01	0.416	−0.025	− 0.77	− 0.035	−1.249	0.024	0.95	0.016	0.506	0.027	1.1
Family income status(RMB/month/per person)(reference: < 2000)												
2000–4999	− 0.189	−6.487^*^	0.08	1.95	0.024	0.677	−0.179	−5.565^*^	− 0.094	−2.385^*^	− 0.188	−6.167^*^
5000–9999	− 0.305	−9.322^*^	0.22	4.803^*^	− 0.105	−2.627^*^	− 0.568	−15.739^*^	− 0.251	−5.655^*^	− 0.374	− 10.955^*^
10,000–19,999	−0.285	−9.7^*^	0.207	5.049^*^	−0.097	− 2.701^*^	− 0.545	− 16.818^*^	− 0.296	−7.446^*^	− 0.357	− 11.646^*^
≥ 20,000	−0.06	−2.958^*^	0.089	3.15^*^	−0.002	−0.087	− 0.11	−4.934^*^	−0.021	− 0.768	− 0.072	−3.394^*^
Payment (reference: free medical treatment)												
Medical insurance	−0.068	− 1.066	− 0.052	− 0.585	− 0.191	− 2.472^*^	− 0.063	− 0.903	− 0.04	− 0.461	− 0.072	− 1.098
New rural cooperative medical insurance	− 0.075	− 1.124	− 0.04	− 0.427	− 0.144	− 1.777	− 0.04	− 0.544	− 0.022	− 0.245	− 0.067	− 0.968
Self-paying	−0.07	− 1.839	− 0.056	−1.045	− 0.076	− 1.63	0.005	0.13	0	− 0.005	− 0.039	− 0.987
Serious disease insurance or commercial Insurance	−0.106	− 2.784^*^	0.024	0.458	−0.144	−3.093^*^	− 0.079	− 1.881	− 0.111	− 2.141^*^	− 0.118	− 2.97^*^
Stage of breast cancer (reference: I)												
II	−0.003	− 0.106	− 0.085	− 2.206^*^	− 0.06	−1.79	0.049	1.615	0.008	0.213	0.012	0.413
III	−0.005	− 0.179	− 0.058	−1.519	− 0.065	− 1.977^*^	0.046	1.517	0.038	1.029	0.005	0.165
IV	0.048	2.088^*^	−0.081	−2.511^*^	− 0.045	− 1.605	0.063	2.47^*^	0.039	1.262	0.049	2.046^*^
Treatment (reference: surgery)												
Surgery + chemotherapy	0.089	2.438^*^	−0.114	−2.219^*^	− 0.049	−1.105	0.056	1.376	0.042	0.843	0.059	1.555
Surgery + radiation therapy	0.012	0.608	−0.015	−0.546	0.007	0.269	0.012	0.52	0.029	1.058	0.015	0.716
Surgery + chemotherapy + radiation therapy	0.139	4.188^*^	−0.1	−2.153^*^	−0.002	− 0.054	0.122	3.343^*^	0.022	0.499	0.1	2.903^*^
Surgery + chemotherapy + radiation therapy + hormone therapy	0.215	6.517^*^	−0.118	−2.556^*^	0.025	0.613	0.151	4.171^*^	0.095	2.123^*^	0.174	5.076^*^
Surgery + targeted therapy	0.095	4.049^*^	−0.074	−2.231^*^	0.025	0.857	0.041	1.589	0.066	2.061^*^	0.08	3.251^*^
Others*	0.133	3.987^*^	−0.05	−1.079	−0.037	− 0.899	0.129	3.494^*^	0.08	1.773	0.111	3.189^*^
Family history of cancer (reference: no)												
Yes	0.052	2.703^*^	−0.047	−1.749	− 0.041	− 1.729	0.016	0.766	0.049	1.866	0.048	2.4^*^
Pain (reference: mild)												
Moderate	0.313	14.452^*^	−0.175	−5.761^*^	0.105	3.959^*^	0.095	3.961^*^	0.134	4.559^*^	0.267	11.798^*^
Severe	0.35	16.404^*^	−0.09	−3.008^*^	0.072	2.784^*^	0.108	4.586^*^	0.131	4.544^*^	0.282	12.665^*^
Physical activity (reference: light)												
Medium	0.011	0.536	0.101	3.63^*^	0.388	16.022^*^	−0.007	−0.318	− 0.006	− 0.232	0.023	1.106
Heavy	0.039	1.988^*^	0.037	1.368	0.166	6.97^*^	0.03	1.375	0.059	2.223	0.066	3.231^*^

^*^:*P* < 0.05

Others*: any other treatment method or combination of surgery, chemotherapy, radiotherapy, targeted therapy, endocrine therapy, or other treatment methods

### Relationship between the CSPro-BC and FACT-B

The Pearson correlation (Table 6) showed that all subscales of the CSPro-BC were negatively associated with all subscales of QoL except the health behaviour domain, with the correlation coefficient ranging from − 0.815 to − 0.011.

**Table 6:** Relationship between unmet needs and QoL

Items	Physical	Social/Family	Emotional	Functional	Additional concerns	Total score FCAT-B
Symptom burden	− 0.790^**^	− 0.072^*^	− 0.674^**^	−0.484^**^	− 0.770^**^	− 0.765^**^
Function	−0.529^**^	−0.380^**^	− 0.538^**^	−0.551	− 0.507^**^	−0.686^**^
Health behavior	−0.368^**^	0.136^**^	−0.203^**^	−0.058^*^	− 0.380^**^	−0.242^**^
Financial strain	−0.559^**^	−0.011^*^	− 0.467^**^	−0.399^**^	− 0.589^**^	−0.558^**^
Healthcare-seeking skills	−0.523^**^	− 0.177^**^	− 0.493^**^	−0.453^**^	− 0.545^**^	−0.602^**^
Total score of CSPro-BC	−0.779^**^	−0.167^**^	− 0.690^**^	−0.558^**^	− 0.777^**^	−0.815^**^

^*^: *P*<0.05, ^**^:*P*<0.01

## Discussion

The identification and management of unmet needs is an essential component of high-quality healthcare for cancer survivors [4]. To our knowledge, this study is the first multicentre cross-sectional report to analyse unmet needs and QoL among Chinese BCSs. The following three aspects will be discussed based on the objectives and results of the research: (i) the unmet needs of BCSs, (ii) factors associated with the unmet needs of BCSs, and (iii) the relationship between the unmet needs and QoL of BCSs in mainland China.

### Unmet needs of BCSs

Findings from this study highlighted that most BCSs faced physical, emotional, and practical concerns after completing treatment. The top 3 unmet needs in this study were in the domains of symptom burdens and healthcare-seeking skills. The level of unmet needs for Chinese BCSs was found to be highest for fear of recurrence, patient-provider communication, and body image. The results of this study are similar to a Korean study that found that the highest level of unmet needs was ‘needed help in coping with fear of recurrence’ [4]. Martínez Arroyo et al. and Skandarajah et al. also reported that needs focused on the possibility of recurrence were the most frequent [17, 18]. However, Chou et al. reported that the most unmet supportive need of BCSs in Taiwan was in the psychosocial domain (40.4%), followed by the nutritional domain (28.4%) and patient care (20.8%), and with the lowest needs concerning the domains of health information, treatment, and finances (11.9, 3.5, and 0.2%) [24].

The healthcare-seeking skills domain of the CSPro-BC ranked second in this study, in which patient-provider communication and health information were found to be highest. Some previous studies [7, 18, 37] showed that the most prevalent unmet needs of BCSs were in the information domain. Even for survivors who are years beyond their diagnosis and treatment, many information needs remain unmet, such as age-appropriate cancer information and information on diet, nutrition, exercise, complementary/alternative healthcare services, assistance with health insurance, mental health counselling, infertility, and religious and spiritual counselling [38].

Body image disturbance is a common problem, resulting from side effects (e.g. alopecia, scars, or loss of breasts) after breast cancer treatment, and is related to poor QoL among BCSs [39]. Because the worry about cancer recurrence, most mainland Chinese women with breast cancer prefer to receive mastectomies [39]. A meta-analysis indicated that women undergoing a mastectomy alone had higher levels of cancer-related distress than those undergoing a mastectomy with immediate or delayed reconstruction [40]. Therefore, aggressive efforts and measures are needed to meet the unmet needs of survivors’ body image disturbances.

### Factors associated with the unmet needs of BCSs

Our study reveals that the unmet needs of BCSs depend on eleven factors, including age, time since diagnosis, education level, occupation, payment, family income status, stage of breast cancer, treatment, family history of cancer, pain, and physical activity. To ensure the provision of high-quality survivorship care and a high satisfaction level, more attention should be paid to actively identifying and addressing the unmet needs of BCSs.

Age is a meaningful variable. This study also revealed that the < 40 age group showed a higher level of recognition of needs in the domains of symptom burden, health behaviour, and financial strain. The ≥50 age group showed fewer unmet needs. This difference can be attributed to the fact that patients who suffer from cancer face both expensive healthcare costs and unemployment stress, the young undertake more family responsibilities in China, and younger BCSs pay more attention to body image. Patients with higher education degree like master degree or above, had more unmet needs in health care-seeking skills. This was contrary to what we used to think that highly-educated patients might more easily acquire medical support. The possible explaination was that highly-educated patients learn more about the disease and they might question the doctor’s advice. Thus, its seems a little difficult to find a suitable physician who meet their high expectations.

In the analysis according to survival time after breast cancer diagnosis, the levels of unmet needs in the domains of symptom burden, function, and health behaviour were higher in the group with a survival time of less than 12 months. Cancer treatment is complicated and time-consuming; those who have survived for less than 12 months have not completed treatment. Therefore, the side effects of treatment and the symptom burden are more serious during this period. Meanwhile, we found that the more therapies that combined, the higher the perceived levels of unmet needs was. Survivors receiving target therapy seem to have more unmet needs and this result is consistent with the findings of Chae et al. [4]. Additionally, patients may not understand the disease or treatment well. A lack of awareness of side effects and disease-related resources also aggravate unmet needs.

In the analysis according to payment, people owned serious disease insurance or commercial insurance method had lower unmet needs in the symptom burdern, health behaviour, and healthcare seeking skills domain. In China, people who buy serious disease insurance or commercial insurance are wealther, thus, there is a high possibility for them to have access to medical resource to release symptom burdern and get more information support.

Side effects after treatment, heavy physical work, poor health and fatigue, depression, and emotional distress entail reduced work engagement and work ability among BCSs [41]. The prevalence of returning to work factors varies from 43 to 93% within 1 year of diagnosis [41]. Therefore, not returning to work and high treatment expenses aggravate the family financial burden. In this study, we found that for families with incomes below 2000 RMB/month have higher levels of unmet needs in all domains.

The analysis of needs according to cancer stage, the levels of unmet needs in the domains of symptom burden, function, health behaviour, and financial strain were higher in the group with advanced stage cancer. Advanced breast cancer patients undergo more complicated treatments, which cause more serious side effects, resulting a greater fear of recurrence and greater difficulty in performing activities of daily life. Other studies have reported the same results [4]. The group with a family history of breast cancer had greater needs, which is probably due to previous experiences with a family member who received breast cancer treatment [4], as well as possible negative attitude to cancer treatment for a sense of ‘unfairness’ for this undeserved illness.

In the analysis of needs according to pain, the levels of unmet needs were higher in all domains in the group with serve pain. Cancer pain is one of the most frequent and disturbing of all cancer-related symptoms [42]. In addition, this is one of the symptoms patients fear most. Pain suffering aggravates physical and symptoms burden [43]. Our study presented that patients with serve pain showed more unmet needs were in all domains, indicating an urgency to relieve pain.

The impaired physical activities was problaly due to fatigue [44], and it was proved that exercise intervention can promote physical functioning and decrease fatigue [45, 46]. In the analysis according to physical activities, the heavier the physical activity, the higher a BCS’s perceived level of unmet needs.

### The relationship between unmet needs and QoL in BCSs

Understanding the relationship between unmet needs and QoL in BCSs is a stepping stone to improving QoL. This study shows that all domains of the CSPro-BC negatively correlate with all subscales of QoL (excluding the health behaviour domain) and overall QoL. BCSs’ QoL is influenced by unmet needs, treatment, disease-related symptom burdens, and psychological stress [21]. Unmet needs are strong predictors of QoL among recurrent breast cancer patients. A pervious study found that the greater the symptom burden that patients faced, the more unmet needs they have and the worse their QoL is [47]. This suggests that the QoL of women with recurrent breast cancer is possibly affected more by unmet needs than by their socio-demographic or clinical characteristics. Therefore, accurate assessment of unmet needs and provision of appropriate services may be an important step in helping survivors attain a high QoL.

### Study limitations

One limitation of this study was its cross-sectional design, as it analysed the unmet needs identified in the CSPro-BC and related factors at only one point in the disease trajectory. Future studies should examine the prospective unmet needs identified in the CSPro-BC and their related factor trajectories at different time points to explore how unmet needs progress across the breast cancer trajectory. In addition, the participants’ Chinese cultural background may limit the generalisability of the results to other targeted populations from different cultures.

### Implications for practice

Despite these limitations, the findings suggest some potential implications for practice. First, this study clarified the specific unmet needs of BCSs in China, related factors, association between unmet needs and QoL, which may be important entry points for intervention. Second, screening patients for unmet needs as early as possible and throughout treatment may be significant in reducing the disease-related burden of BCSs. Finally, the results offer a basis for deriving improved treatment outcomes and the grounds for providing comprehensive care for BCSs. Healthcare professionals should aim to detect patients’ unmet needs as early as possible and provide cancer survivorship care to BCSs to improve their QoL.

## Conclusion

The Chinese BCSs in this study exhibit a high level of unmet needs, the most prevalent of which were found in the symptom burden domain. There was a significant association between patients’ unmet needs and QoL. Future research should focus on addressing unmet needs, the implementation of earlier interventions for emerging concerns, enhancing survivorship or follow-up care, and further improving the QoL of BCSs.

## Data Availability

The datasets used and analyzed during the current study are available from the corresponding author on reasonable reason.

## Abbreviations

BCS: Breast cancer survivors
FCR: Fear of cancer recurrence
CSPro-BC: Cancer Survivor Profile:Breast Cancer
FACT-B: Functional Assessment of Cancer Therapy-Breast
QoL: Quality of life
